# Experimental Assessment of Possible Factors Associated with Tick-Borne Encephalitis Vaccine Failure

**DOI:** 10.3390/microorganisms9061172

**Published:** 2021-05-29

**Authors:** Ksenia Tuchynskaya, Viktor Volok, Victoria Illarionova, Egor Okhezin, Alexandra Polienko, Oxana Belova, Anastasia Rogova, Liubov Chernokhaeva, Galina Karganova

**Affiliations:** 1 FSBSI “Chumakov FSC R&D IBP RAS”, 108819 Moscow, Russia; kseniya-tuchka@mail.ru (K.T.); viktor.p.v@mail.ru (V.V.); illarionova_vv@chumakovs.su (V.I.); ohezin_ev@chumakovs.su (E.O.); polienko_ae@chumakovs.su (A.P.); mikasusha@bk.ru (O.B.); rogova_aa@chumakovs.su (A.R.); dec151ll@mail.ru (L.C.); 2 Department of Biology, Lomonosov Moscow State University, 119991 Moscow, Russia; 3 Institute of Translational Medicine and Biotechnology, Sechenov First Moscow State Medical University, 119991 Moscow, Russia

**Keywords:** tick-borne encephalitis, TBEV, flavivirus, vaccine failure, mouse model, cyclophosphamide, immunosuppression, non-infectious virus particles, structural heterogeneity, neuroinvasion

## Abstract

Currently the only effective measure against tick-borne encephalitis (TBE) is vaccination. Despite the high efficacy of approved vaccines against TBE, rare cases of vaccine failures are well documented. Both host- and virus-related factors can account for such failures. In this work, we studied the influence of mouse strain and sex and the effects of cyclophosphamide-induced immunosuppression on the efficacy of an inactivated TBE vaccine. We also investigated how an increased proportion of non-infectious particles in the challenge TBE virus would affect the protectivity of the vaccine. The vaccine efficacy was assessed by mortality, morbidity, levels of viral RNA in the brain of surviving mice, and neutralizing antibody (NAb) titers against the vaccine strain and the challenge virus. Two-dose vaccination protected most animals against TBE symptoms and death, and protectivity depended on strain and sex of mice. Immunosuppression decreased the vaccine efficacy in a dose-dependent manner and changed the vaccine-induced NAb spectrum. The vaccination protected mice against TBE virus neuroinvasion and persistence. However, viral RNA was detected in the brain of some asymptomatic animals at 21 and 42 dpi. Challenge with TBE virus enriched with non-infectious particles led to lower NAb titers in vaccinated mice after the challenge but did not affect the protective efficacy.

## 1. Introduction

Tick-borne encephalitis virus (TBEV) is the cause of one of the most serious viral diseases damaging the central nervous system (CNS) in people across Europe and Asia. There are three main subtypes of TBEV: Siberian, European and Far Eastern [1]. Several distinct divergent groups of TBEV have also been distinguished [2]. Despite the genetic distance between the TBEV subtypes, they have significant serological cross-reactivity [3].

The only effective measure against tick-borne encephalitis (TBE) is prophylactic vaccination. A number of formaldehyde-inactivated whole-virion vaccines against TBE is approved worldwide in adult and pediatric formulations [4]. Two vaccines are available in Europe, namely FSME-Immun (Baxter, Wien, now Pfizer, Austria) and Encepur (Novartis, Marburg, Germany). These vaccines are based on the European TBEV strains K23 and Neudorfl, accordingly. Several other vaccines are licensed in Russia: Tick-E-Vac and its lyophilized analog, referred to as TBE vaccine Moscow (FSBSI “Chumakov FSC R&D IBP RAS”, Moscow, Russia), and EnceVir (Microgen, Tomsk, Russia), which are based on the Far Eastern TBEV strains Sofjin and 205, respectively [5,6,7]. A vaccine used in China (SenTaiBao, Changchun Institute of Biological Products Co., Ltd., Jilin, China) is based on the Sen-Zhang strain (Far Eastern TBEV subtype) [8]. Vaccination against TBE has been proven effective, as exemplified by the results of mass vaccination campaigns in Austria [9] and Russia [10,11,12,13]. Randomized controlled trials in large populations have shown the induction of strong antibody response and low incidence of adverse events after TBE vaccination [1,14,15,16,17,18]. In animal experiments it has been shown that vaccination against TBE induces protective titers of neutralizing antibodies (NAb) to heterologous TBEV subtypes [5,19,20]. In humans, vaccination by the licensed vaccines provides high antibody titers against all main virus subtypes [3,20,21,22,23,24].

However, rare cases of TBE vaccine failures have also been recorded [4,25,26,27,28,29]. Cases of breakthrough TBEV infections in vaccinated people are usually characterized by a mild clinical course [25], but severe and even lethal infections have also been described [4,27,28,29]. The purported explanation given by the authors for this is antibody dependent enhancement of infection (ADE), which is well known for mosquito-borne flaviviruses such as dengue and Zika viruses. However, ADE in the case of dengue virus infection is observed upon re-infection with a heterologous virus subtype, which often co-circulate [30]. Reported cases of TBE vaccine failure were registered on the territories where the virus subtype corresponded to the vaccine TBEV subtype, such as in Europe and the Far East of Russia. It also cannot be ruled out that mild TBE cases after vaccination are more difficult to register, which may affect the observed prevalence of the disease [31]. Nevertheless, whether the phenomenon of ADE is relevant to TBEV infection is unknown, and clear evidence for it is lacking [29,31].

The causes of the vaccine failures can be associated with the characteristics of the host [32]. Such factors as the genetic predisposition to TBEV infection [33], sex, age, body mass index, immune status and comorbidities at the time of vaccination or at the time of infection [24,25,34,35,36], and impaired vaccination schedule were also investigated in relation to the vaccine failures [37]. The efficacy of vaccination against several infections has been shown to be age-dependent, and the vaccine failures were usually associated with older age [38]. In addition to the host-related and vaccine-related factors, possible features of the virus itself could also increase the probability of breakthrough infections. Such factors as the infection dose of the virus, its virulence, reproduction rate, route of inoculation, etc. were shown to influence the severity of the disease and the immune response [39].

Potentially, structural heterogeneity of the virus should also be taken into account when studying the vaccine failures. Flavivirus virions are characterized by a significant structural diversity. An ensemble of particles is produced during flavivirus infection, including mature infectious virions, immature and partially mature virions, the envelope protein E aggregates [40,41,42,43,44], and dead-end noninfectious structures formed as a result of short-term exposure to increased temperatures [45]. Each of these structures carries the protein E on its surface in a variety of conformational positions, however, they are partially or completely non-infectious, and thus can interfere with the infectious virions in functional assays, affect the assessment of the effective concentration of antivirals targeting the protein E [46,47] or immune response.

Neuroinvasion of the virus is an important factor determining the outcome of TBEV infection. After entering the CNS, viral infection can lead either to a lethal disease, complete elimination of the virus, or to the development of a persistent (chronic) infection. For TBE, chronic forms of the disease are registered mainly in Russia, which probably is a feature of the Far Eastern and Siberian TBEV subtypes [1,48]. The possibility of persistent TBE infection in rodents has been established as well. It is known from animal experiments that TBEV can persist in the CNS of several species of wild rodents, which do not develop a symptomatic disease [49,50], or in the brain of susceptible laboratory mice surviving the infection [51]. Still, the mechanisms and factors influencing the neuroinvasion of TBEV and its prolonged persistence in the CNS are not clear. Importantly, it is also unknown whether the vaccination is able to completely protect from TBEV neuroinvasion, considering the fact that it does not provide sterilizing immunity and allows for some replication of the virus [52].

Since experimental challenge studies of vaccines against lethal infections are possible only in animal models, a valid, robust and reproducible animal model of infection is essential. The most common model used for studying the protective efficacy of TBE vaccines is susceptible mice (usually, of the strain BALB/c). Different mouse strains are known to have varying susceptibility to flaviviruses, including TBEV [53,54,55], which could influence the efficacy of tested vaccines. However, it is currently unknown whether this model is suitable for investigating the factors that contribute to the vaccine failures in humans.

Thus, in our work, we attempted to study the efficacy of an inactivated TBE vaccine in mice focusing on such parameters of the host as the mouse strain and sex and to assess the influence of transient immunosuppression on the vaccine efficacy. In addition, we investigated how the ratio of non-infectious to infectious viral particles in the virus sample used for challenge affected the protective response to TBE vaccination. The vaccine efficacy was analyzed in terms of immunogenicity and protection against death, disease symptoms and persistence of TBEV in the CNS of vaccinated animals 3–6 weeks after infection.

## 2. Materials and Methods

### 2.1. Animals

Susceptible inbred mice of the strain BALB/c and outbred ICR mice (State Institution Scientific Center of Biotechnology, branch “Stolbovaya”, Moscow, Russia) were used in this study. The particular strain, average age and sex of mice are given in the Results section for each experiment. The animals were kept and treated in accordance with the international recommendations for the treatment of laboratory animals (CIOMS recommendations, 1985, the Directive 2010/63/EU, and Appendix A to the European Convention ETS No. 123). Bioethics committee of FSBSI “Chumakov FSC R&D IBP RAS (protocol #17 from 1 September 2016) approved all experimental procedures performed on animals.

### 2.2. Cells and Viruses

Porcine embryo kidney (PEK) cell line was maintained at 37 °C in medium 199 with Hanks’ balanced salt solution and Earle’s balanced salt solution (2:1, *v*:*v*, FSBSI “Chumakov FSC R&D IBP RAS”, Moscow, Russia) supplemented with 5% fetal bovine serum (FBS, Invitrogen, Waltham, MA, USA). Vero cell line was maintained at 37 °C in DMEM medium supplemented with L-glutamine (FSBSI “Chumakov FSC R&D IBP RAS”, Moscow, Russia) and 10% FBS (Invitrogen, Waltham, MA, USA).

Several TBEV strains belonging to the Far Eastern, European and Siberian TBEV subtypes were used for the virus challenge (Table 1). The particular strain and dosage of virus sample are given in the Results section for each experiment.

The fresh virus passage in the mouse brain were obtained from intracerebrally (i/c) infected suckling mice (3–4 days old) and collected when severe neurological signs of the disease manifested, and the cell culture fluid 48 h post infection was collected when the first signs of cytopathic effect appeared. The virus was stored as aliquots at −70 °C prior to use.

### 2.3. Infection of Ticks

Five *Ixodes ricinus* ticks from the laboratory culture were infected percoxally according to the previously described technique [56] with TBEV Absettarov strain in the dosage of 3.3–3.6 log PFU/tick. After the infection, ticks were kept in glass tubes with gradient humidity at room temperature (23 °C). On day 7 after the infection ticks were frozen at −70 °C for further virological analysis.

### 2.4. Vaccination and Cyclophosphamide (cy) Treatment

In all experiments we used an inactivated vaccine Tick-E-Vac based on TBEV strain Sofjin (“Chumakov FSC R&D IBP RAS” Moscow, Russia). The vaccine was injected intramuscularly (i/m) in the volume of 50 μL per mouse (1:10 of the human dose) into the hind thigh muscle. In all experiments we used two-dose vaccination schedule with 14-day intervals both between two vaccine doses and the viral challenge. Control mice were twice i/m inoculated with 50 µL of saline on corresponding days.

To achieve transient immunosuppression, cyclophosphamide solution (Cy) (Endoxan, Baxter, Westfalen, Germany) was injected intraperitoneally (i/p) at two dosage schemes (low-dose and high-dose treatments). The first scheme consisted of two i/p injections with one day interval at doses of 60 mg/kg and 30 mg/kg, respectively. BALB/c mice were divided into four experimental groups: the first group was immunized twice with a subsequent s/c challenge with 100 LD_50_ of TBEV strain Vasilchenko (Vas) (hereafter named “Vac-Vac” for brevity); the second group was treated with Cy one day before the first vaccine dose (“Cy-Vac-Vac”); and the third group was vaccinated twice and treated with Cy immediately before the challenge (“Vac-Vac-Cy”). The last group received no treatment before the virus challenge and served as a control (“Virus”).

For the high-dose treatment, the first injection dose was 80 mg/kg, and the second dose administered with one day interval was 40 mg/kg. Independent of the scheme, the second injection in both experiments was administered one day before the vaccination or the virus challenge. Cy-Vac-Vac group was treated with Cy before the first vaccination; in Сy-Vac-Cy-Vac group the immunosuppressive treatment was carried out before the first vaccine dose and repeated before the second vaccine dose; Vac-Cy-Vac group received Cy only before the second vaccination; also, one group of mice was vaccinated only once (“Vac”). First control group was once again vaccinated twice and left without immunosuppression (“Vac-Vac”), while the second control group was left intact before the challenge (“Virus”).

The low-dose Cy treatment was adjusted for 3–4 week-old BALB/c mice and did not lead to mortality, but caused transient weight loss and lymphopenia (Figures S1 and S2). The high-dose scheme led to mortality of 17% of animals and to more pronounced weight loss and lymphopenia (Figures S1 and S2). Blood samples were taken from separate groups of mice receiving the Cy treatment (n = 2–3) and the white blood cell (WBC) were counted in the hemocytometer.

### 2.5. Plaque Assay and 50% Plaque Reduction Neutralization Test (PRNT_50_)

Plaque assay was performed as described earlier [47] on PEK cell monolayers in 24-well plates. Serial 10-fold dilutions of TBEV were incubated with PEK cells for 1 h at 37°C in a CO_2_-incubator, coated with 1.26% methylcellulose (Sigma, St Louis, MO, USA) in medium 199 with Hanks’ and Earle’s salts (2:1, *v*:*v*) and 2% FBS, and left for incubation for 6 days at 37 °C in a CO_2_-incubator. Then the cells were fixed with 96% ethanol and stained with 0.4% gentian violet dye. Infectious virus titer was expressed in log PFU/mL. Each sample was titrated in at least three biological replicates in several independent experiments.

Plaque reduction neutralization test (PRNT_50_) was performed on PEK cell monolayers in 24-well plates. Mouse blood samples were collected from the submandibular vein [57], serum was separated from the clots by centrifugation and stored in aliquots at −20°C. For the test, 5-fold dilutions of sera from individual mice in medium 199 with 2% FBS were incubated with TBEV samples (1:1, *v*:*v*) in the concentration of 30–40 PFU per well for 1 h at 37 °C. Virus-serum mixture (100μL) was added to PEK cells in 24-well plates and incubated for 1 h. The proceeding steps were identical to the procedure described for the plaque assay. Every experiment included controls, i.e., negative and positive murine sera with known antibody titers. The NAb titers were calculated according to the modified Reed and Muench method [58].

### 2.6. Virus Titration in Mice

For the quantification of 50% lethal dose of TBEV, 8-week-old BALB/c mice in groups of five were injected subcutaneously (s/c) with 100 μL of 10-fold dilutions of the virus in medium 199 and observed for clinical symptoms and mortality for 21 days [59]. Ruffled fur, hunched posture, limb paresis and paralysis, and the body weight loss were considered disease symptoms. The lethal dose of virus resulting in 50% mortality (expressed in log LD_50_) was calculated according to the Kerber method [60].

### 2.7. Sample Preparation and RNA Quantification (qRT-PCR)

Brain samples of sacrificed mice were weighed and manually homogenized in medium 199 to obtain 10% suspensions, which were aliquoted and stored at −70 °С. Total RNA was isolated from the brain suspension according to the protocol described earlier [47]. A fixed amount (5.5 log RNA copies per sample) of attenuated type I Sabin poliovirus vaccine strain was added to each sample as an internal control [61,62]. For the primers see Table S1. Quantitative RT-PCR (qRT-PCR) was performed according to a modified method described earlier [47,62] on Dyad Disciple thermal cycler with a Chromo4 detector (BioRad, Hercules, CA, USA), using Taq polymerase, RealTime PCR kit and primers (Synthol, Moscow, Russia). TBEV RNA levels were measured in duplicates and expressed as log of viral RNA equivalents per ml of the sample after comparison with a standard prepared as described in [63]. The limit of detection for this method was determined by serial dilutions to be 50 TBEV RNA copies per PCR reaction tube (or 1000 RNA equivalents per ml of the initial sample). Taking into account the dilution factor and the linear range of the standard curve, the limit of quantification was set as 1000 RNA copies per PCR reaction (2 × 10^4^ copies per mL of the sample). RNA quantity in the virus samples was expressed as logarithm of genome containing particles (GCP) per ml (log GCP/mL).

### 2.8. Statistical Analysis

Statistical analyses were performed using GraphPad Prism 9 (GraphPad Software Inc., San Diego CA, USA). Differences in morbidity and mortality were assessed using Fisher’s exact test. Median survival time (MST) and incubation period (IP) were presented as median and range and compared by Mann-Whitney U-test. For survival analysis, Kaplan-Meier curves were plotted and analyzed by the log rank test with a Bonferroni correction for multiple comparisons. Viral RNA burden in the brain, NAb levels, viral titers and GCP/PFU ratios were compared by Mann-Whitney U-test.

## 3. Results

### 3.1. Host-Related Factors of the Vaccine Failure in a Mouse Model

#### 3.1.1. Influence of Mouse Strain and Sex on the Susceptibility to TBEV Infection and Efficacy of TBE Vaccine

**Figure 1:**
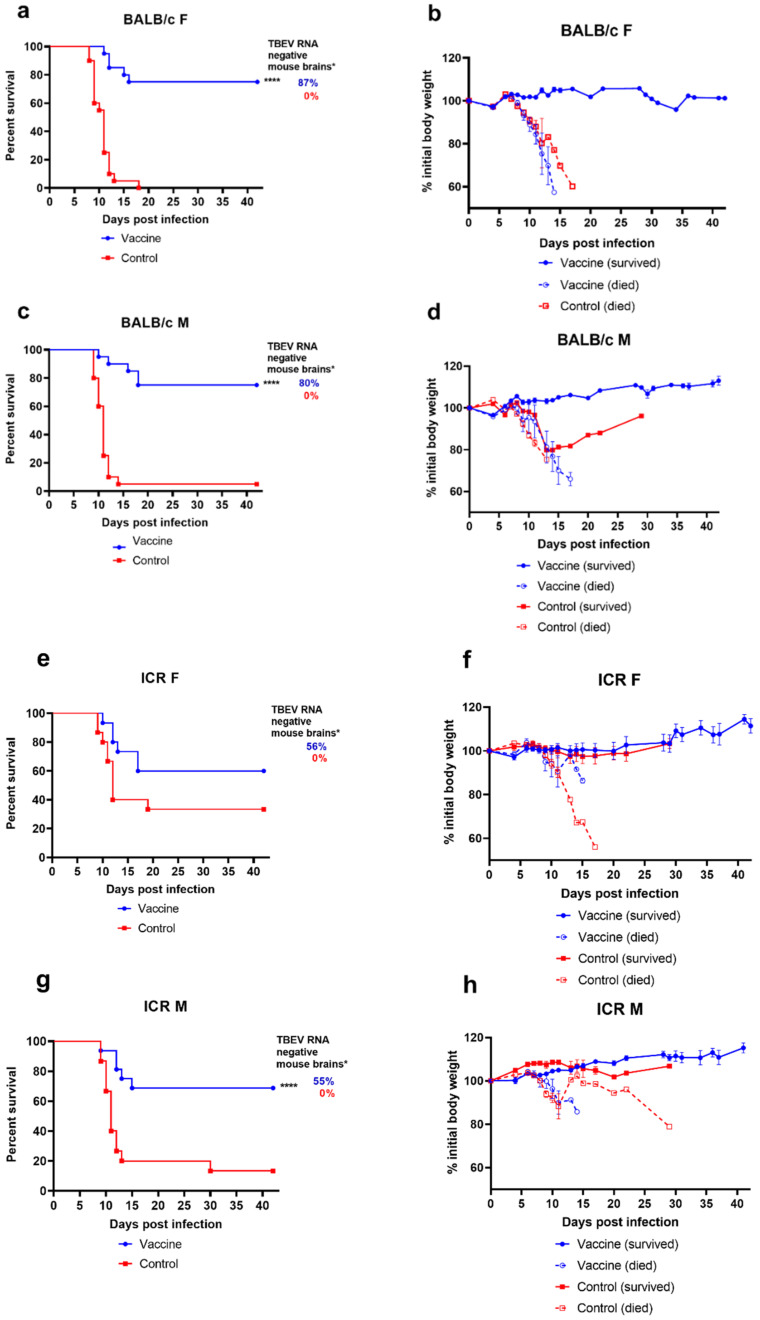
Survival and weight changes in BALB/c and ICR mice vaccinated with TBE vaccine Tick-E-Vac after challenge with 300 PFU/animal of TBEV strain Vasilchenko. (**a**,**b**) female BALB/c mice (n = 20 per group), (**e**,**f**) female ICR mice (n = 15 per group), (**c**,**d**) male BALB/c mice (n = 20 per group), (**g**,**h**) male ICR mice (n = 16 for vaccine group, n = 15 for control group). Animals were monitored for mortality (**a**,**c**,**e**,**g**), weight loss (**b**,**d**,**f**,**h**), and disease symptoms (data not shown) for 42 days after challenge. Error bars represent SEM. * All brain samples from dead mice were considered RNA-positive. Statistical significance for mortality data was determined by log rank test (**** *p* < 0.0001).

In this experiment, we compared the efficacy of vaccination in inbred BALB/c and outbred ICR mice, which are more genetically diverse. Mice of 3–4 months of age were divided into four groups according to their strain and sex (BALB/c male, BALB/c female, ICR male, ICR female). To assess the protective efficacy of vaccination, we registered the levels of mortality, morbidity (as prolonged weight loss and/or clinical symptoms), and the presence or absence of viral RNA in the brain of surviving animals. For the challenge, mice were s/c inoculated with TBEV strain Vas in a dose of 300 PFU/mouse. After the challenge, mice were observed for 42 days (Figure 1), then sacrificed, and brain samples from each animal were tested for viral RNA content, as specified in Materials and Methods.

First, we compared the susceptibility of non-vaccinated mice of all four groups to TBEV infection. For BALB/c, the mortality was 100% and 95% of female and male control animals, respectively. Male ICR mice had 87% mortality, which was not statistically different from BALB/c mice (*p* > 0.05, Fischer *t*-test), while female ICR mice were significantly less susceptible to the virus with 60% mortality by the end of the observation period (*p* < 0.01). No difference in morbidity, MST or IP was observed between any of the control groups. Thus, BALB/c mice of both sexes and ICR males were more susceptible to TBEV, than female ICR mice.

Vaccination protected both male and female BALB/c mice and male ICR mice against disease symptoms (Figure 1b,d,h) and death (69–75% of vaccinated mice in these groups survived compared to almost complete mortality in control groups, *p* < 0.0001) (Figure 1a,c,g). In contrast, the vaccine-induced protection was much worse for the least susceptible female ICR mice (more than 50% developed some symptoms and 40% died in the vaccine group, while 93% of non-vaccinated mice had disease symptoms, but only 67% died (Figure 1e,f)).

Among vaccinated BALB/c mice of both sexes some animals that developed disease symptoms recovered by the end of observation period, while animals that developed a lethal disease still had a slightly longer IP and MST (Table S2) (*p* < 0.01 and *p* < 0.05, correspondingly, Mann-Whitney test). This was not the case for vaccinated male and female ICR mice, among which all animals that got sick eventually succumbed to the disease. There were no statistically significant differences in IP and MST between sick ICR male mice in vaccinated and control groups (Table S2) (*p* > 0.05, Mann-Whitney test). In contrast to BALB/c mice, for which all disease symptoms, weight loss and death occurred within 21 days of observation (standard time span for TBE infection), in the group of more heterogeneous ICR mice, weight loss and subsequent death for was observed in some animals at later days (21–30 dpi, Figure 1h).

Next, we assessed the levels of TBEV RNA in brains of all surviving mice from vaccinated and control groups at 42 days post infection. Naturally, we considered all died animals as TBEV RNA-positive. The vaccination protected all groups of mice against neuroinvasion and persistence of the viral RNA, although the level of protection was incomplete. TBEV RNA was found in 100% of brain samples of surviving non-vaccinated mice, and in 28% of brains of vaccinated animals.

Although most vaccinated BALB/c mice had no detectable TBEV RNA (80–87% RNA negative) (Figure 1a,c and Figure S3). Notably, viral RNA was detected in the brain samples of some asymptomatic animals. Some vaccinated mice from BALB/c F, BALB/c M and ICR F groups exhibited symptoms of disease, but no viral RNA was detected in their brains at day 42, and most vaccinated mice with detectable RNA levels were clear of any symptoms (Figure S3).

The RNA levels were significantly higher in female and male non-vaccinated ICR mice than in vaccinated groups (*p* = 0.004 and *p* = 0.015, correspondingly (Mann-Whitney test)), and the highest amounts of RNA (>6 log RNA copies/mL) were detected in non-vaccinated mice with obvious symptoms (Figure S3).

#### 3.1.2. Effect of Immunosuppression on TBE Vaccine Efficacy in BALB/c Mice

**Figure 2:**
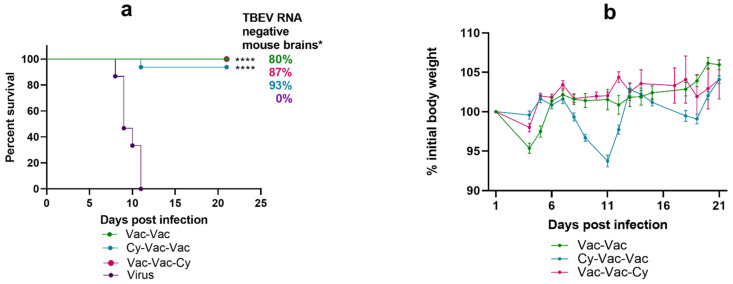
Survival and weight changes in BALB/c mice with and without low-dose Cy immunosupression. Mice were vaccinated with TBE vaccine Tick-E-Vac and challenged with 100 LD_50_ of TBEV strain Vasilchenko. (**a**) Survival curves for vaccinated and control mice. (**b**) Weight changes after the virus challenge. Error bars represent SEM. Group Vac-Vac was vaccinated twice without Cy treatment (green); group Cy-Vac-Vac received Cy before the first vaccine dose (blue); group Vac-Vac-Cy received Cy after the vaccination and immediately before the challenge (pink); Virus—a control non-vaccinated group (violet). In all vaccinated groups, the vaccine was i/m administered twice (50 mkl/mouse) with a two-week interval; the virus challenge was carried out two weeks after the second vaccination. Cy was i/p administered twice at doses 60mg/kg and 30 mg/kg with a one-day interval. For all groups, N = 15. Data only for mice surviving till 21 dpi was used for weight curves. * All brain samples from dead mice were considered RNA-positive. Statistical significance for mortality data was determined by log rank test (**** *p* < 0.0001).

First, to model a mild immunosuppression, we used the low-dose Cy treatment scheme (see Materials and Methods). In this experiment, the vaccine demonstrated robust protection under immunosuppressive conditions. Vaccination protected 93–100% of mice against death and provided 80–90% protection against disease symptoms and neuroinvasion at 21 dpi in all three vaccinated groups (Figure 2, Table S3). Compared to the control group (Vac-Vac), in the immunosuppressed group Cy-Vac-Vac animals lost body weight on 8–13 dpi, which coincided with the acute phase of the disease (Figure 2b).

**Figure 3:**
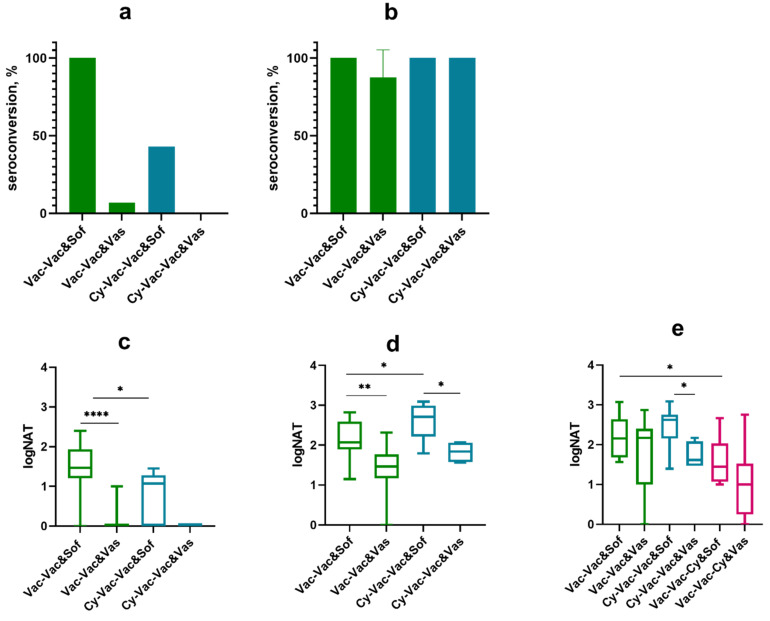
Seroconversion and levels of neutralizing antibodies (NAb) against TBEV vaccine strain Sofjin (&Sof) and the challenge strain Vasilchenko (&Vas) in sera of Cy-treated mice (low-dose scheme). (**a**) Seroconversion rate after the first vaccine dose and (**b**) after the second vaccine dose. NAb titers were measured in sera of mice (**c**) 10 days after the first vaccine dose, (**d**) 10 days after the second vaccine dose, and (**e**) one day after the challenge with Vas TBEV strain. The control group Vac-Vac did not receive Cy treatment (green); Cy-Vac-Vac group was treated with Cy before the first vaccine dose (blue); Vac-Vac-Cy was treated with Cy after two-dose vaccination and immediately before the challenge (pink). N = 7–8 mice per group. In (**a**–**d**) Vac-Vac group includes the data for Vac-Vac and Vac-Vac-Cy groups, since at this stage of the experiment they received identical treatment. * *p* < 0.05; ** *p* < 0.01; **** *p* < 0.0001.in Mann-Whitney test. Box plots indicate median and range.

In Vac-Vac and Vac-Vac-Cy groups the first vaccination provided 100% seroconversion against strain Sofjin KGG (Sof) with NAb titers >1 log, while in Cy-Vac-Vac group the seroconversion level was less than 50%, with significantly lower antibody levels (*p* < 0.05) (Figure 3a,b). Almost no animals had measurable NAb titers against Vas TBEV strain after a single vaccine dose (Figure 3с). However, after the second vaccination, NAb titers in all groups reached >1 log against both TBEV strains with almost 100% seroconversion rate (Figure 3b,d). Still, NAb titers against Vas strain were significantly lower than against Sof (Figure 3d).

One day after the virus challenge, NAb titers in groups Vac-Vac and Cy-Vac-Vac approached the level of 2 log against both TBEV strains, but were significantly lower in Vac-Vac-Cy group (*p* < 0.05), which received Cy immediately before the challenge (Figure 3e). The main difference between NAb titers against strain Sof and Vas (*p* < 0.05) were found in Cy-treated group Cy-Vac-Vac (Figure 3e), which suggests that the treatment with Cy has narrowed the range of antibody response to TBEV in vaccinated animals.

On day 14 after Cy treatment, we observed an increase in WBC count (Figure S1a). This data was consistent with an increase in NAb titers after the second dose of vaccine in the Cy-Vac-Vac group (Figure 3d).

**Figure 4:**
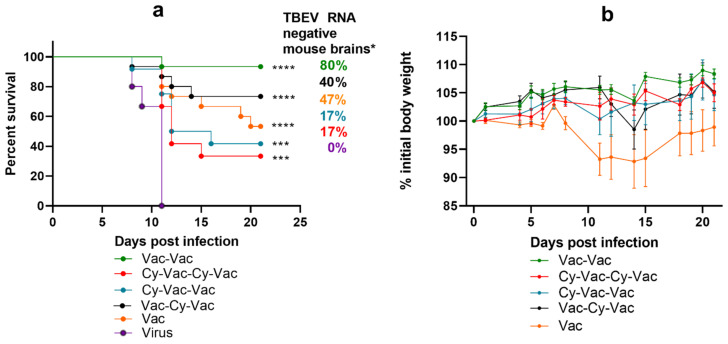
Survival and weight changes in BALB/c mice with and without high-dose Cy immunosupression. Mice were vaccinated with TBE vaccine Tick-E-Vac and challenged with 100 LD_50_ of TBEV strain Vasilchenko. (**a**) Survival curves for vaccinated and control mice. (**b**) Weight changes after the virus challenge. Error bars represent SEM. Vac-Vac group was used as a control without Cy treatment (green); Cy-Vac-Vac received Cy treatment before the first vaccine dose (blue); Cy-Vac-Cy-Vac was treated with Cy twice: before the first and the second vaccine doses (red); Vac-Cy-Vac received Cy treatment before the second vaccine dose (black) and Vac was vaccinated only once (orange). N = 12–15 animals per group. Data only for mice surviving till 21 dpi was used for weight curves. * All brain samples from dead mice were considered RNA-positive. Statistical significance for mortality data was determined by log rank test (*** *p* < 0.001; **** *p* < 0.0001).

We increased the number of groups and used a higher dose of Сy to test how various timing of more pronounced immunosuppression would affect the vaccine efficacy (Figure 4a).

As expected, the largest number of vaccine failure cases was observed in the group treated with Cy both before the first and the second vaccination (Cy-Vac-Cy-Vac, (Figure 4a)). Nevertheless, the vaccine provided partial protection against mortality (33–73%), low protection against disease (17–60%), and even lower protection against TBEV neuroinvasion and persistence in the CNS at 21 dpi (17–47%) (Figure 4a,b, Table S4) in Cy-treated groups (Figure 4a,b).

**Figure 5:**
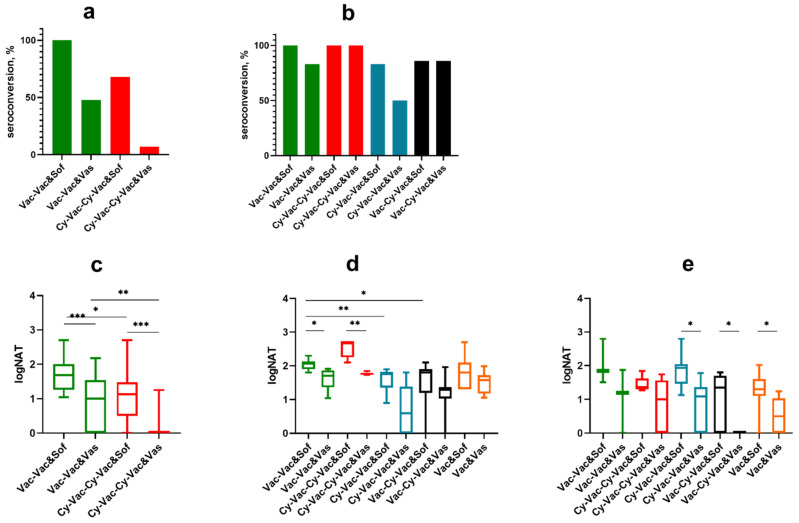
Seroconversion and levels of neutralizing antibodies against TBEV strains Sofjin (&Sof) and Vasilchenko (&Vas) in sera of Cy-treated mice (high-dose scheme). (**a**) Seroconversion rate after the first vaccine dose and (**b**) the second vaccine dose. Nab titer (**c**) after the first vaccine dose, (**d**) the second vaccine dose, and (**e**) one day after the challenge with 100 LD_50_ TBEV strain Vasilchenko: Vac-Vac group was used as a control without Cy treatment (green); Cy-Vac-Vac received Cy treatment before the first vaccine dose (blue); Cy-Vac-Cy-Vac was treated with Cy twice: before the first and the second vaccine doses (red); Vac-Cy-Vac was treated with Cy before the second vaccine dose (black); and Vac was vaccinated only once (orange). In (a) and (c) Vac-Vac group includes the data for Vac-Vac, Vac-Cy-Vac and Vac groups, Cy-Vac-Cy-Vac includes the data for Cy-Vac-Vac group since at this stage of the experiment they received identical treatment. N = 7–8 animals per group. * *p* < 0.05; ** *p* < 0.01; *** *p* < 0.001 obtained by Mann-Whitney test. Box plots indicate median and range.

In this experiment seroconversion rates on day 10 after the first vaccination against vaccine strain Sof reached 100% only in groups that did not undergo any immunosuppression at this time, while in groups that received pretreatment with Cy seroconversion was 68% (Figure 5a). There was a significant difference in NAb titers against two TBEV strains between the control group Vac-Vac and the groups treated with Cy (Figure 5с). As in the previous experiment, seroconversion rates and NAb titers against strain Vas were significantly lower after a single vaccine dose in all groups (seroconversion 0–30%, NAb titers <1 log, *p* < 0.01 (Figure 5a)). After the second vaccination, NAb titers increased in all studied groups, however the titers against strain Vas were lower than against Sof, especially in Cy-treated groups (Cy-Vac-Vac and Vac-Cy-Vac), where the immunosuppression was more pronounced (Figure 5d). On the next day after the challenge with strain Vas, we did not see significant differences in NAb titers against strain Sof between the groups, but antibody levels against strain Vas were significantly lower than against strain Sof in Cy-Vac-Vac, Vac-Cy-Vac and Vac groups (Figure 5e).

### 3.2. Virus-Related Factors of the Vaccine Failure in Mouse Model

We investigated the influence of the ratio between non-infectious and infectious virus particles (log (GCP/PFU)) on the vaccine efficacy. To obtain TBEV samples with an increased content of non-infectious virus particles, we simulated different storage conditions for the EK-328 strain samples (Figure S4a,b). This was based on an earlier observation that for some samples that were stored for more than 20 years and/or were thawed and freezed several times, the GCP/PFU ratio was greater than for others (Figure S5). The largest portion of non-infectious viral particles (~5 log) was observed for the virus sample heated for 48 h at 37 °C.

An equal dose expressed in LD_50_ of the heated (+37 °C for 48 h) and intact (untreated) TBEV strain EK-328 were used to challenge female BALB/c mice in the vaccine efficacy experiments. During the observation, there were no differences in MST and IP parameters between non-vaccinated mice infected with heated or intact virus, although there was a tendency to an increase of IP for the group challenged with the heated virus (*p* = 0.08) (Table S5). The presence of a large number of non-infectious viral particles did not affect the vaccine efficacy (100% of vaccinated animals survived).

**Figure 6:**
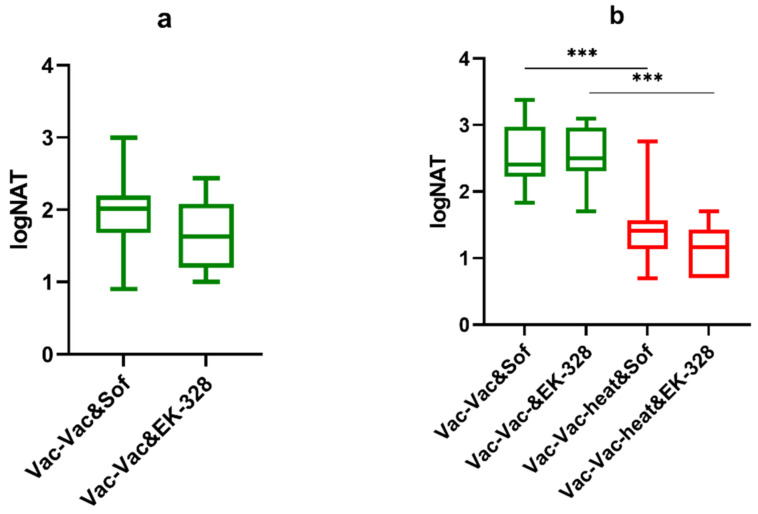
NAb titers in vaccinated BALB/c mice against two TBEV strains Sofjin and EK-328 (**a**) after the second vaccine dose (**b**) on day 2 after the virus challenge with TBEV EK-328 strain (green) (n = 7–8) (Vac-Vac) and TBEV EK-328 strain heated at 37 °C for 48 h (Vac-Vac-heat), red) (n = 7–8). The virus infection dose was equalized to 30 LD_50_ per mouse for both groups. *** *p* < 0.001 obtained by Mann-Whitney test. Box plots indicate median and range.

The second vaccine dose provided good induction of antibodies in both groups (Figure 6a, Vac-Vac group) against strains Sof and EK-328 (Figure 6a). In the control group challenged with the untreated virus, we observed a booster response characterized by a significant rise in NAb titers after the challenge. On the contrary, in the group challenged with the heated virus (Vac-Vac-heated), we observed a significant decrease in NAb titers against both TBEV strains Sof and EK-328 (*p* = 0.009 and *p* = 0.012 for NAb titers against Sof and EK-328 respectivly) (Figure 6b, Vac-Vac group) in comparison to the NAb titers after the second vaccination (Figure 6a,b).

To assess the possible strain-related differences in GCP/PFU ratio, we calculated the number of infectious and non-infectious viral particles in the samples of TBEV strains Sof, Vas, EK-328, and Absettarov (Abs), belonging to three different TBEV subtypes with different passage histories and different systems of reproduction at the last passage. Table 2 shows the amounts of PFU and GCP per 100 LD_50_ of TBEV samples of different strains. Numbers of PFU ranged from 100 to 130,000 per 100 LD_50_, while the GCP amounts varied significantly more (from 1000 to 1,000,000 per 100 LD_50_).

Here, we used four TBEV strains: EK-328, Abs, 256 and Sof. Fresh virus passages of each strain were obtained in two reproduction systems: suckling ICR mouse brain and PEK cell culture. Strain Abs was also injected in *I. ricinus* ticks and reproduced for 7 days.

**Figure 7:**
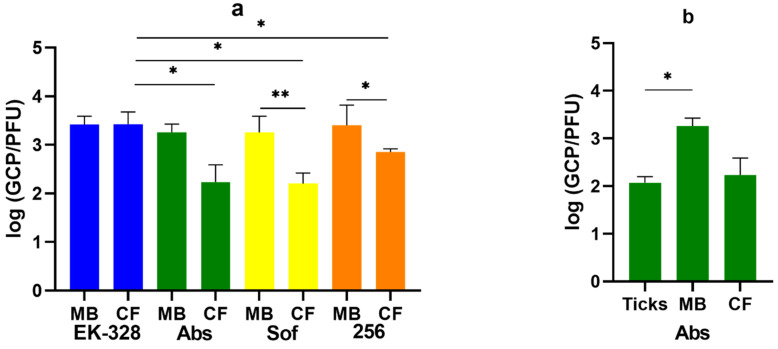
The values of log (GCP/PFU) for the virus samples of four TBEV strains: EK-328 (blue), Absettarov (Abs, green), Sofjin (Sof, yellow) and 256 (orange) after the reproduction (**a**) in PEK cell culture (CF) and in ICR suckling mouse brains (MB) after i/c inoculation. (**b**) GCP/PFU ratio of Absettarov TBEV strain after 7 days in *Ixodes ricinus* ticks. The experiment was carried out in 3–5 biological replicates. Error bars indicate SD. * *p* < 0.05; ** *p* < 0.01 obtained by Mann-Whitney test.

Significant differences in the GCP/PFU ratio were observed between strains EK-328 and strains Abs (*p* = 0.035), Sof (*p* = 0.015), and 256 (*p* = 0.035) after their reproduction in the cell culture (Figure 7a). There were no statistical differences in GCP/PFU ratio after reproduction of these strains in the mouse brain. Thus, for strains Abs, Sof and 256 the GCP/PFU ratio was different after one passage in the cell culture and in suckling mouse brains (*p* = 0.023, *p* = 0.009, and *p* = 0.035, respectively) (Figure 7a). The GCP/PFU ratio also differed for the strain Abs after passages in *I. ricinus* ticks and in the mouse brains (*p* = 0.023 Mann-Whitney test) (Figure 7b). Thereby, the ratio between GCP and PFU can be different both between various TBEV strains and between viruses obtained in different systems of reproduction.

## 4. Discussion

The efficacy of inactivated vaccines against TBE has been established by a significant amount of experimental, clinical, and epidemiological data obtained through decades of their use across Europe and Asia. Still, TBE cases among vaccinated, although rare, are registered on a regular basis.

Despite extensive research on antiviral vaccines and pathogenesis of viral infections, the causes of such breakthrough infections are not clear for even the more studied diseases. Generally, vaccine failures can be associated with either the individual characteristics of the host, vaccine-related factors (e.g., vaccine quality, vaccination schedule, time between the last vaccine dose and infection), or the properties of the virus (such as the dose, virus strain, and others).

Human studies aimed to assess the vaccine efficacy are usually retrospective in nature, and the search of eligible subjects is complicated. In addition, it is rather difficult to take into account all the factors influencing the vaccine failure in humans due to the population heterogeneity. Considering the limited nature of studies with human subjects, we evaluated the suitability of a laboratory mouse model as the most extensively utilized animal model of flavivirus encephalitis, for studying the vaccine failure.

We collected and analyzed several standard parameters to characterize the experimental TBEV infection in vaccinated mice, including mortality, disease symptoms, MST, IP, induction of NAb and presence of viral RNA in the CNS at 21 or 42 dpi. Detection of viral RNA in the brains of infected and vaccinated mice was especially important for neurotropic TBEV flavivirus with a potential ability to persist in the CNS of various species, including humans.

Epidemiological evidence suggests that the likelihood of vaccine failure is related to the age, sex, and genetic makeup of the host. While there are some studies showing sex-related differences in susceptibility of mice to flavivirus infections [64], the data on the role of sex as a predisposing factor to TBE in mice are limited. It is known that different strains of mice have varying susceptibility to TBEV [53,54,55], but there is no data on the strain-related differences in the efficacy of vaccination, so we chose the strain used most commonly, BALB/c, and compared it to a more genetically diverse outbred ICR mice. In our experiments, we saw that mouse strain and sex could be the factors affecting both susceptibility to the virus and the vaccine efficacy. For BALB/c mice there were virtually no differences nor in susceptibility to TBEV infection nor in the protective efficacy of the vaccine between males and females. On the contrary, female ICR mice were less susceptible to the virus and at the same time relatively less protected by the vaccination after TBEV infection than male ICR mice. 

Only 28% of vaccinated surviving mice had some amounts of TBEV RNA at 42 dpi, and the RNA levels were significantly lower than in control animals. The absence of viral RNA in the brain of mice that exhibited disease symptoms at some point indicates that the virus either could have been completely eliminated from the CNS or that its amount decreased to undetectable levels. Titration or passaging of such RNA-negative brain samples in the cell culture did not reveal any infectious virus (data not shown). This suggests that the vaccine provided protection to BALB/c and ICR mice against neuroinvasion and persistence of TBEV, although this protection was not complete. Thus, it is important to distinguish two parameters that characterize such animal models, which are the susceptibility to infection and the efficacy of vaccination against this infection, although they are likely to be connected. Further research in this direction would help to elaborate the immunological and pathophysiological mechanisms determining both of these characteristics and their interactions.

Another possible host-related cause of TBE vaccine failures could be immunosuppression of various origins. Notably, the use of immunosuppressants, such as methotrexate or TNF-inhibitors before or during the course of vaccination was shown to negatively affect TBE vaccine efficacy in humans [34]. It is possible that immunosuppression could impair the efficacy of the vaccine if experienced in the period before, during or after the infection by TBEV-infected tick bite, although there is no direct data supporting this hypothesis. In order to address these issues, we simulated transient immunosuppression in a mouse model by injections of cyclophosphamide (Cy) at different stages of the vaccination with inactivated TBE vaccine and before the virus challenge. Cy is one of the most widely used cytotoxic and immunosuppressive agents prescribed for treatment of various cancers and autoimmune disorders [65]. Its action is mainly directed to suppress the B-cell immune response [66]. It has only a partial effect on the regulatory T cells [67,68]. Cy is also actively used as an immunosuppressant in various mouse models [69].

In our experiments the low-dose Cy-induced immunosuppression (which by itself did not cause significant weight loss or death) affected the levels and spectrum of antibodies induced by the vaccine and almost did not affect the protective efficacy, i.e., levels of death, disease, and viral RNA persistence in the CNS. In contrast, the more severe immunosuppression caused by a higher dose of Cy led to death of some animals before the virus challenge, changed the NAb spectrum after the virus challenge, and reduced the protective efficacy of the vaccine by all studied parameters. Proportion of mice with persisting TBEV at 21 dpi ranged from 20% in intact vaccinated mice to 83% in mice receiving two high-dose Cy treatments before and after the first vaccination. Thus, we observed a correlation between the degree of immunosuppression, levels of disease symptoms and death, and viral persistence in the CNS. We observed the lowest vaccine efficacy (comparable to a single immunization) in the groups which received Сy treatment before the first vaccine dose (Cy-Vac-Vac and Cy-Vac-Cy-Vac groups). Accordingly, even a mild immunosuppression affected the spectrum of the antibody response. It could be significant because our data showed that in the context of immunosuppression high titers against the vaccine virus strain may not always be sufficient to protect against the challenge virus.

We also assessed several virus-related factors that could lead to TBE breakthrough infections. The differences in the vaccine efficacy against various TBEV strains can be explained by several reasons, including high virulence of a particular strain [70], features of the interaction of different strains with the immune system [39], and genetic [71,72] or structural heterogeneity of the virus sample. It was previously shown that the vaccine efficacy can be associated with the properties of the virulent virus and, probably, with its antigenic structure [59]. In this work, we used two TBEV strains belonging to Siberian subtype for the virus challenge. We showed that NAb titers in mouse sera after two-dose vaccination were sufficient to provide protection against heterologous TBEV strains, which agrees with the previous studies [59]. In comparison to the vaccine strain Sofjin, the NAb titers against the Vasilchenko strain at all stages of vaccination were significantly lower than against EK-328 strain, which indicates that there are important differences in the features of TBEV strains belonging to the same subtype.

The main direction of our research is emphasized by the fact that TBEV population is comprised of a wide variety of structurally different viral particles, which could affect both the development of the immune response during flavivirus infection [73] and the outcomes of the virus challenge studies. Moreover, the number of non-infectious viral particles in the studied TBEV strains exceeded the number of infectious viral particles 100 to 1000 times, as was shown before [47,74]. It is known for mosquito-borne flaviviruses that the number of non-infectious immature virus particles may depend on the reproduction system or individual characteristics of the strain [75]. It has been shown that prolonged incubation of virions at 37 °C led to the formation of “dead-end” non-infectious viral particles [44]. In our experiments we used analogous conditions (incubation at 37 °С for 48 h) to enrich the TBEV samples with non-infectious particles. In addition, our results indicate that the amount of non-infectious viral particles in TBEV samples may depend on the duration and conditions of the storage of the virus samples. Prolonged storage in the freezer, exposure to ambient temperatures, slight heating or repeated freeze-thaw cycles led to loss of infectivity of the samples, but did not affect the GCP numbers, effectively enriching the viral population with non-infectious particles.

In this work, we demonstrated that the presence of a large proportion of non-infectious particles in TBEV samples used for the challenge after vaccination affected the booster antibody response to infection but did not reduce the vaccine efficacy. The surface of flavivirus virions is extremely heterogeneous and contains many different epitopes [44,76,77]. Non-infectious viral particles can play a role not only in the induction of the immune response [45,78,79], but also act as antibody decoys and affect the vaccine efficacy. As can be seen from our data, a large proportion of non-infectious particles decreased the booster effect after the virus challenge, which could lead to the vaccine failure. In addition, the ratio of infectious to non-infectious particles depended on the virus strain and the system of reproduction. This suggests that the virus variants circulating in nature may differ in this characteristic. Therefore, to obtain valid results, virus samples used for in vivo and in vitro experiments should be characterized and standardized for the ratio of non-infectious to infectious viral particles.

In conclusion, the main factor that ensures the vaccine efficacy is the booster antibody effect, i.e., a rapid increase in the levels of specific antibodies after the infection with a virulent virus. Obviously, many factors can influence this antibody booster. Our work revealed several possible reasons for the booster NAb decrease. Firstly, even a slight immunosuppression of the host before the vaccination, and secondly, the structural heterogeneity of the challenge virus sample both led to significant decrease of the booster response. In our experiments, this decrease did not result in antibody levels falling below the protective levels, although different TBEV strains differed in their ability to be neutralized by vaccine-induced antibodies. However, under certain conditions, all of these factors or their combination could lead to the vaccine failure.

## Figures and Tables

**Table 1:** TBEV strains used for virus challenge and in Plaque Reduction Neutralization Test (PRNT_50_).

TBEV Strain	Region and Year of Isolation	Source of Isolate	Passage History *	GenBank Accession Number
***Far Eastern Subtype***				
SofjinKGG	Primorsky Krai, 1937	Brain of deceased TBE patient	МхР1М3P1	GU121963
***Siberian subtype***				
Vasilchenko	Novosibirsk region, 1961	Blood of TBE patient	МхМ2V1	L40361
EK-328	Estonia, 1972	*I. persulcatus* tick*s*	M6P1M6P2	DQ486861
***European Subtype***				
256	Belarus, 1940	*I. ricinus* tcks	MxM6	AF091014
Absettarov	Leningrad region, Russia, 1951	blood of a TBE patient	MxM5	KU885457

* M—mouse brain passage (Mx—unidentified number of early passages before the virus was obtained by the laboratory); P—passage in PEK cell culture, V—passage in Vero cell culture.

**Table 2:** Amounts of GCP and PFU per 100 LD_50_ of different TBEV samples.

TBEV Strain	Passage History *	LD_50_	PFU	GCP
	*Far Eastern Subtype*			
Sofjin KGG	P1M2	100	130,000	350,000
	*Siberian Subtype*			
Vasilchenko	хМ2V1	100	5,000	60,000
EK-328	М6P1М4		100	1000
	*European Subtype*			
Absettarov	Mx	100	10,000	1,000,000

* M—mouse brain passage (Mx—unidentified number of early passages before the virus was obtained by the laboratory); P—passage in PEK cell culture, V—passage in Vero cell culture.

## Data Availability

Data is contained within the article or supplementary material.

## Supplementary Materials

The following are available online at https://www.mdpi.com/article/10.3390/microorganisms9061172/s1, Figure S1: number of leukocytes in the peripheral blood of mice (a) after the low-dose Cy treatment and (b) after the high-dose Cy treatment on days 0, 7 and 14, Figure S2: weight changes before the virus challenge (day 28), Figure S3: TBEV RNA levels on 42 dpi in brains of surviving mice vaccinated with TBE vaccine Tick-E-Vac the challenge with 300 PFU of Vasilchenko TBEV strain, Figure S4: the values of log (GCP/PFU) for the samples of PEK cell culture fluid containing TBEV strain EK-328 at different storage conditions, the values of log (GCP/PFU) for the virus samples of three TBEV strains, Table S1: primers and probes, Table S2: protective activity of Tick-E-Vac in ICR and BALB/c mice against TBEV strain Vasilchenko, Table S3: protective efficacy of Tick-E-Vac vaccine against TBEV strain Vasilchenko in BALB/c mice treated with Cy before or after the vaccination, Table S4: protective activity of Tick-E-Vac vaccine in BALB/c mice treated with Cy against TBEV strain Vasilchenko, Table S5: protective activity of Tick-E-Vac vaccine in BALB/c mice against TBEV heated and unheated virus strain EK-328.
