# Supplementary material for: Experimental Assessment of Possible Factors Associated with Tick-Borne Encephalitis Vaccine Failure
Source: Microorganisms. 2021 May 29;9(6):1172. doi: 10.3390/microorganisms9061172 (PMC8229799; doi:10.3390/microorganisms9061172)
Supplement: Supplementary file 1 [file microorganisms-09-01172-s001.zip › microorganisms-1232902-supplementary.pdf]

Supplementary Information

Experimental assessment of possible factors associated with TBE vaccine failure

Ksenia Tuchynskaya[1], Viktor Volok[1,2], Victoria Illarionova[1,2], Egor Okhezin[1,2], Alexandra Polienko[1], Oxana Belova[1], Anastasia Rogova[1], Liubov Chernokhaeva[1], Galina Karganova[1,3]*

FSBSI “Chumakov FSC R&D IBP RAS”, Moscow 108819, Russia; sue_polio@chumakovs.su

[2] Department of Biology, Lomonosov Moscow State University, Moscow 119991, Russia; info@mail.bio.msu.ru

[3] Institute of Translational Medicine and Biotechnology, Sechenov First Moscow State Medical University, Moscow 119991, Russia; rektorat@sechenov.ru

* Corresponding author. Email: karganova@bk.ru

Supplementary Table 1. Primers and probes

Target virus	Primer name	Sequence	Target genome region
TBEV	TBE/Pow3’	5’-AGCGGGTGTTTTTCCGAGTC-3’	3’-NTR of TBEV
	F-TBE1	5’-GGGCGGTTCTTGTTCTCC-3’	
	TBE-Probe	5’-(FAM)-TGAGCCACCATCACCCAGACACA-(BHQ1)-3’	
	R-TBE1	5’-ACACATCACCTCCTTGTCAGACT -3’	
Poliovirus	PVL1	5’-GGCAGACGAGAAATACCCAT -3’	3Dpol of poliovirus
	PVR1	5’-CGAACGTGATCCTGAGTGTT-3’	
	PVP1	5’-(R6G)-TTGATTCATGAATTTCCTTCATTGGCA-(BHQ1)-3’	

Supplementary table 2. Protective activity of Tick-E-Vac in ICR and BALB/c mice against TBEV strain Vasilchenko.

Group	N	Vaccination	IP, median (range), days	MST, median (range), days	Survived animals, %	Healthy animals, %
BALB/c F	20	+	8 (8 — 30)	12 (11 — 16)	75	65
	20	-	7,5 (7 — 11)	11 (9 — 18)	0	0
BALB/c M	20	+	9 (8 — 12)	17 (10 — 18)	75	60
	20	-	8 (6 — 9)	11 (9 — 14)	5	0
ICR F	15	+	9 (7 — 31)	12,5 (10 — 17)	60	47
	15	-	9 (6 — 11)	11,5 (8 — 19)	33	7
ICR M	16	+	8 (7 — 16)	13 (9 — 13)	69	62
	15	-	9,5 (7 — 16)	11 (9 — 30)	13	0

MST – median survival time; IP – incubation period;

Supplementary table 3. Protective efficacy of Tick-E-Vac vaccine against TBEV strain Vasilchenko in BALB/c mice treated with Cy before or after the vaccination.

Group	N	IP, median (range), days	MST, median (range), days	Survived animals, %	Healthy animals, %	RNA negative mouse brains*, %
Virus	15	7 (7-9)	9 (8-11)	0	0	0
Virus-Cy	15	7 (6-9)	10 (8-11)	0	0	0
Vac-Vac-Cy	15	-	-	100	87	87
Cy-Vac-Vac	15	-	-	93	80	93
Vac-Vac	15	-	-	100	80	80

MST – median survival time; IP – incubation period;
* All animals that died from the disease were counted as RNA-positive.

Supplementary table 4. Protective activity of Tick-E-Vac vaccine in BALB/c mice treated with Cy against TBEV strain Vasilchenko.

Group	N	IP, median (range), days	MST, median (range), days	Survived animals, %	Healthy animals, %	RNA negative* mouse brain, %
Virus	15	7.1	10.1	0	0	0
Cy-Vac-Cy-Vac	12	8.5 (8-14)***	11.5 (11-15)*	33	17	17
Cy-Vac-Vac	12	8.0 (6-11)	12.0 (8-16)**	42	25	17
Vac-Cy-Vac	15	9.0 (7-15)**	11.5 (8-14)**	73	60	40
Vac	15	11.0 (7-14)***	12.0 (11-20)**	53	33	47
Vac-Vac	15	-	-	93	93	80

MST – median survival time; IP – incubation period;
* All animals that died from the disease were counted as RNA-positive .

* $p<0.05$; ** $p<0.01$ and ***$p<0.001$ significance compared to virus group obtained by Mann-Whitney test

Suplementary table 5. Protective activity of Tick-E-Vac vaccine in BALB/c mice against TBEV heated and unheated virus strain EK-328.

Group	N	IP, median (range), days	MST, median (range), days	Survived animals, %	Healthy animals, %
Virus	15	9.0 (10-13)	13.0 (15-17)	0	0
Virus+37	15	13.0 (8-14)	15.5 (13-19)	0	0
Vac-Vac-Vir	15	-	-	100	100
Vac-Vac-Vir+37	15	-	-	100	100

MST – median survival time; IP – incubation period;

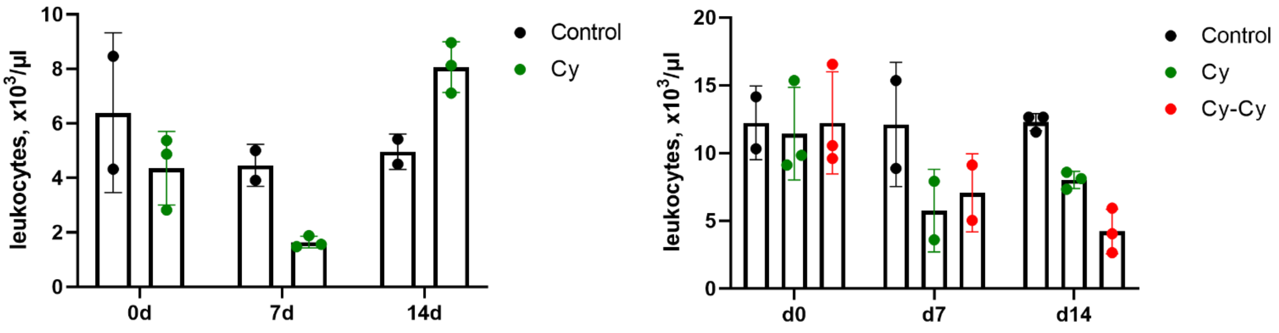

Figure S1. Number of leukocytes in the peripheral blood of mice (a) after the low-dose Cy treatment and (b) after the high-dose Cy treatment on days 0, 7 and 14. N=2-3 animals per group.

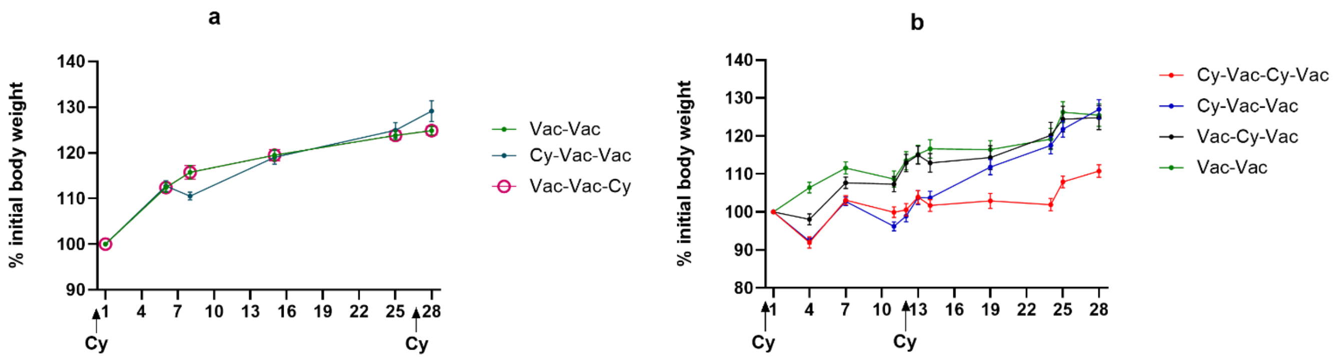

Figure S2. Weight changes before the virus challenge (day 28). Two vaccine doses were administrated at day 3 and 16. a) Graph for the low-dose Cy experiment that included three groups: Vac-Vac was vaccinated twice without Cy treatment (green); Cy-Vac-Vac received Cy before the first vaccine dose (blue); Vac-Vac-Cy received Cy after the vaccination and immediately before the challenge (pink); b) Graph for the high-dose Cy experiment that included four groups: Vac-Vac was a control group (green) without Cy treatment; Cy-Vac-Vac received Cy treatment before the first vaccine dose (blue); Cy-Vac-Cy-Vac was treated with Cy twice (before the first and the second vaccine doses) (red); Vac-Cy-Vac received Cy treatment before the second vaccine dose (black). N=12-15 animals per group. Error bars represent SEM

Groups that did not receive two vaccine doses are not shown in the graphs.

Data only for mice surviving till 28 day post treatment with Cy was used for weight curves.

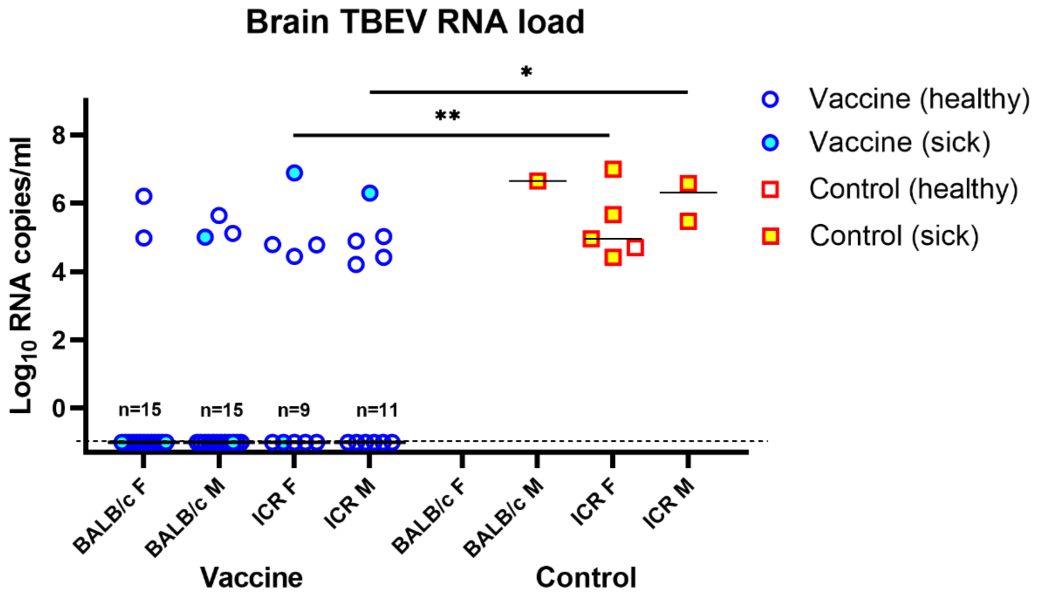

Figure S3. TBEV RNA levels on 42 dpi in brains of surviving mice vaccinated with TBE vaccine Tick-E-Vac the challenge with 300 PFU of Vasilchenko TBEV strain. Viral RNA level is presented as log RNA copies per ml of 10% brain suspension. For the sample preparation and RT-qPCR details see Materials and Methods. n – total number of surviving mice per group. Colored data points (cyan for vaccinated, yellow for control animals) represent individual animals exhibiting some disease symptoms during the observation period. Median values are shown by horizontal lines. Statistical significance was determined by Mann-Whitney test, *$p<0.05$; ** $p<0.01$.

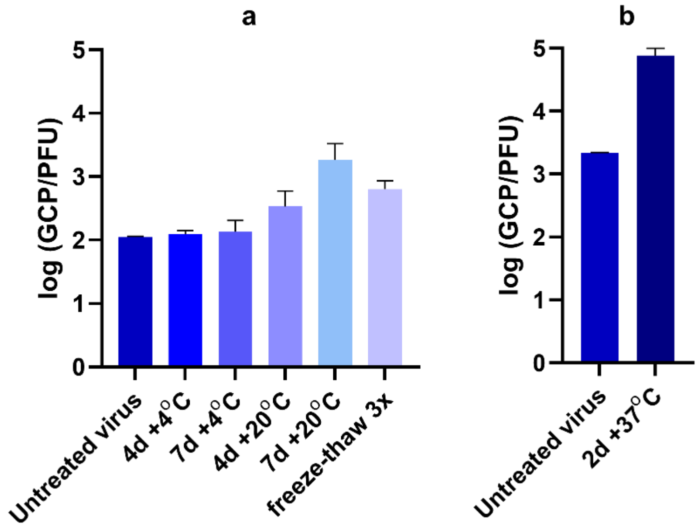

Figure S4. The values of log (GCP/PFU) for the samples of PEK cell culture fluid containing TBEV strain EK-328 at different storage conditions: (a) 4 days at +4˚C, 4 days at +20˚C; 7 days at +4˚C, and 7 days at +20˚C; freezed-thawed 3 times; (b) a separate sample of the same virus was placed at +37˚C for 2 days. Untreated virus was obtained from the fresh passage in PEK cells, frozen at -70 ˚C and used for preparation of other samples immediately after thawing. Samples were quantified by qRT-PCR and plaque assay in at least 3 replicates. Error bars represent SD.

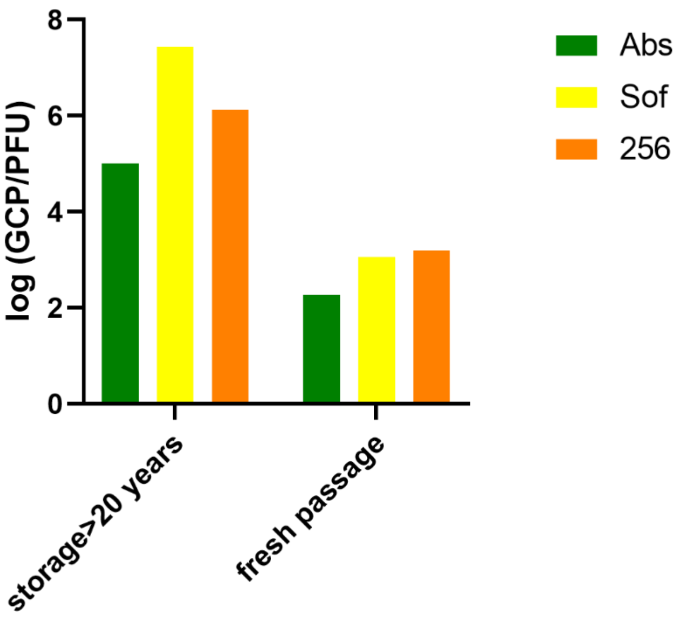

Figure S5. The values of log (GCP/PFU) for the virus samples of three TBEV strains (Absettarov (green), Sofjin (yellow), and 256 (orange)) after their reproduction in PEK cell culture and subsequent storage for less than 5 years at -70˚C and with unknown storage condition for over 20 years.
